# Free of choice on anterior and posterior chest tube position after lung cancer resection

**DOI:** 10.1093/icvts/ivac069

**Published:** 2022-03-14

**Authors:** Qiang Pu, Jian Zhou, Quan Zheng, Jianqi Hao, Dongsheng Wu, Ruoxi Zhang, Hang Wang, Tengyong Wang, Lunxu Liu

**Affiliations:** 1 Department of Thoracic Surgery, West China Hospital, Sichuan University, Chengdu, China; 2 Western China Collaborative Innovation Center for Early Diagnosis and Multidisciplinary Therapy of Lung Cancer, Sichuan University, Chengdu, China; 3 West China School of Medicine, Sichuan University, Chengdu, China; 4 Institute of Thoracic Oncology, West China Hospital, Sichuan University, Chengdu, China

**Keywords:** Chest tube insertion, Pleural drainage, Video-assisted thoracic surgery, Postoperative complications

## Abstract

**OBJECTIVES:**

The optimal location to insert a chest tube for postoperative drainage has not been identified. We performed a retrospective equivalence study to identify whether the efficiency is similar regarding anterior or posterior position of chest tube in thoracic cavity after video-assisted thoracoscopic surgery for non-small-cell lung cancer.

**METHODS:**

A retrospective review of 4263 patients undergoing non-small-cell lung cancer resection from October 2009 to August 2019 in the Western China Lung Cancer Database was conducted. Propensity score matching was performed to balance baseline characteristics between anterior and posterior groups. Chest tube duration, drainage volume, postoperative complications and hospitalization cost were compared. Equivalence margin was defined as (−1, 1) in 95% confidence interval of the mean difference of chest tube duration.

**RESULTS:**

After propensity score matching, we investigated 2912 patients with anterior or posterior (1456 vs 1456) chest tube location following lung cancer resection. The mean time to chest tube removal was 3.39 days in the anterior group and 3.38 days in the posterior group (*P* = 0.52), while the mean difference and 95% confidence interval were 0.02 (-0.17, 0.20). The mean postoperative hospital stays in 2 groups were 5.47 vs 5.24 days (anterior vs posterior, *P* = 0.02). No significant differences were identified regarding the drainage volume during the first 3 postoperative days, postoperative complications and hospitalization cost.

**CONCLUSIONS:**

The comparison of clinical outcomes between anterior and posterior location of chest tube met the criteria for equivalence. For lung cancer patients undergoing video-assisted thoracoscopic surgery resection, it was free choice on anterior or posterior single-tube insertion.

**Figure:**
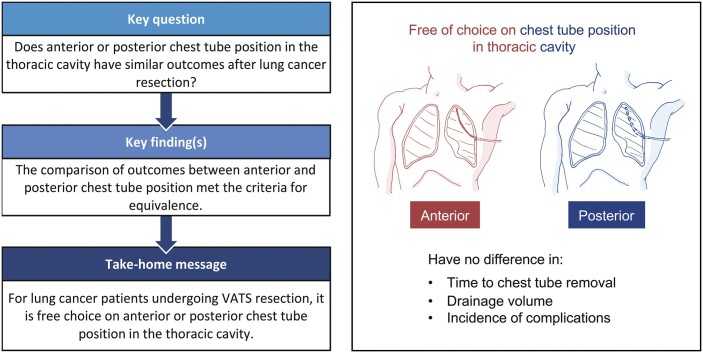

## INTRODUCTION

Chest drainage is a routine procedure during the recovery of almost all lung resection surgery. Following lung resection surgery, the formation and accumulation of fluid and air in pleural cavity may induce dyspnoea, pneumonia or emphysema, therefore delaying recovery [1, 2]. Postoperative chest drainage could eliminate excessive fluid and air to accelerate rehabilitation after lung surgery. Efforts to improve the chest drainage have attracted more and more attention [3, 4]. Video-assisted thoracoscopic surgery (VATS) for non-small-cell lung cancer resection has been gradually accepted as the mainstream surgical approach for operable patients [5, 6]. Optimized perioperative managements play an essential role in improving clinical outcomes of VATS. Although various regimens have been applied to improve drainage, we have not reached a consensus on the position inside the thoracic cavity to place chest tubes. We hypothesized that the efficiency of chest tube is similar whether to insert anteriorly or posteriorly in the thoracic cavity following lung resection.

## PATIENTS AND METHODS

### Ethical statement

Ethic approval has been obtained from the Institutional Ethic Committee for Clinical Research of West China Hospital, Sichuan University [no. 2019 (287)]. Patients’ written informed consent was waived.

### Patient selection

We searched patients undergoing lung cancer surgery via VATS from October 2009 to August 2019 in the Western China Lung Cancer Database, a prospectively maintained database at the department of Thoracic Surgery, West China Hospital. We included patients who: (i) underwent non-small-cell lung cancer resection with VATS; (ii) used single tube for chest drainage; and (iii) the position of chest tube stayed still through postoperative chest radiology inspection. We excluded patients who: (i) had a history of cardio-thoracic surgery; (ii) had a history of tuberculosis that could cause more pleural effusion; and (iii) had conversion to thoracotomy.

### Surgical and post-surgical procedures

All the procedures were performed by the same medical team to reduce excessive impacts on the final result. Patients undergoing VATS for lung cancer resection were enrolled. Before closing the chest wall, a single 28F chest tube (Yangzhou Hanjiang Huafei Medical Device Factory, Co., Ltd, Hanzhou, China) was inserted to the anterior or posterior of thoracic cavity through the existing port site and then connected to water-seal bottle without negative pressure. The decision on position of tube was based on surgeons’ preferences and experiences. The depth of chest tube chest tube inserted in thoracic cavity is about 20 cm. The tip of tube was placed in an apical position. There was 1 hole by each 5 cm on the tube. The patients were instantly transferred to the postanaesthesia recovery unit and then to the ward after consciousness was regained. A chest radiology was conducted routinely to examine lung expansion and chest drainage on the first postoperative day and after chest tube removal. Two authors independently identified the positions (anterior group or posterior group) of chest tubes based on the lateral chest radiology. The drainage volume was recorded every day until the removal of chest tube. The removal criteria included: (i) <300 ml drainage fluid/day; (ii) no bubbling was observed 12 h after clamping the chest tube; and (iii) adequate lung reinflation in chest radiology.

### Data collection

Data were collected on demographics, smoking history, comorbidities, preoperative pulmonary function, surgery details, pathological stage, postoperative drainage and postoperative complications. The 8th edition of the Union for International Cancer Control and the American Joint Committee on Cancer TNM Staging System was used to assess the pathological stage [7]. The primary outcomes comprised of the time to chest tube removal. The secondary outcome variables were the drainage volume within the first 3 postoperative days, the total drainage volume, the length of postoperative hospital stay, incidence of postoperative complications and hospitalization cost.

### Sample size estimation

We estimated the sample size using PASS (11.0.3, NCSS) based on chest tube duration in an equivalence design. The equivalence margin was defined as −1 to 1 in 95% confidence interval (CI) of mean difference. We assumed the true difference as 0.83 and standard deviation of differences as 1.5 according to previous studies analysing postoperative chest tube duration [8, 9]. The type I error rate was set as 5% and statistical power as 99%. On the basis of the equivalence test from a paired design, the sample size was computed to be 1229:1229.

### Statistical analysis

Continuous variables were presented as mean ± standard deviation and compared using Student’s *t*-test before matching and paired *t*-test after matching. Categorical variables were given as count and proportions and compared using the Chi square test before matching and McNemar test after matching. All tests were two-sided and results were considered significant when *P* < 0.05. According to our prior studies involving postoperative chest drainage [8–10], the equivalence was defined as the 95% CI of the mean of differences of the chest tube duration lying within −1 to 1 days. Statistical analyses were performed using R 4.0.2 (R Development Core Team, Vienna, Austria). We further conducted subgroup analyses in the matched cohort, regarding uniportal or three-portal VATS, individual surgeons and resection extent. As a sensitivity analysis, we performed a propensity score methodology with inverse probability of treatment weights (IPTWs). The IPTWs were estimated with multinomial logistic regression analysis, using variables the same as those in the above propensity score-matched (PSM) analysis.

### Propensity score-matched analysis

To generate 2 groups (anterior and posterior group) with comparable baseline characteristics, we conducted PSM analysis. The variables used in PSM included demographic characteristics [age at surgery, sex, body mass index, smoking status], predicted forced expiratory volume in 1 s, predicted diffusion capacity of carbon oxide, pathological maximum tumour diameter, tumour histology, pathological stage, resection extent, resected lobes, surgeons, pleural adhesion and development of intralobular fissure. PSM pairs were identified using a 1:1 nearest greedy neighbour matching algorithm without replacement and with calliper width according to the recommendation from Austin (0.2 of the standard deviation of the logit of the propensity scores) [11]. We assessed the balance of covariates between the 2 groups with the absolute standardized mean difference before and after the matching procedure. An absolute standardized mean difference of ≤0.1 indicated balance in covariates between the 2 groups [12]. The PSM was performed using ‘matchit’ package in R 4.0.2 (R Development Core Team, Vienna, Austria).

## RESULTS

### Patients’ baseline characteristics

**Figure 1:**
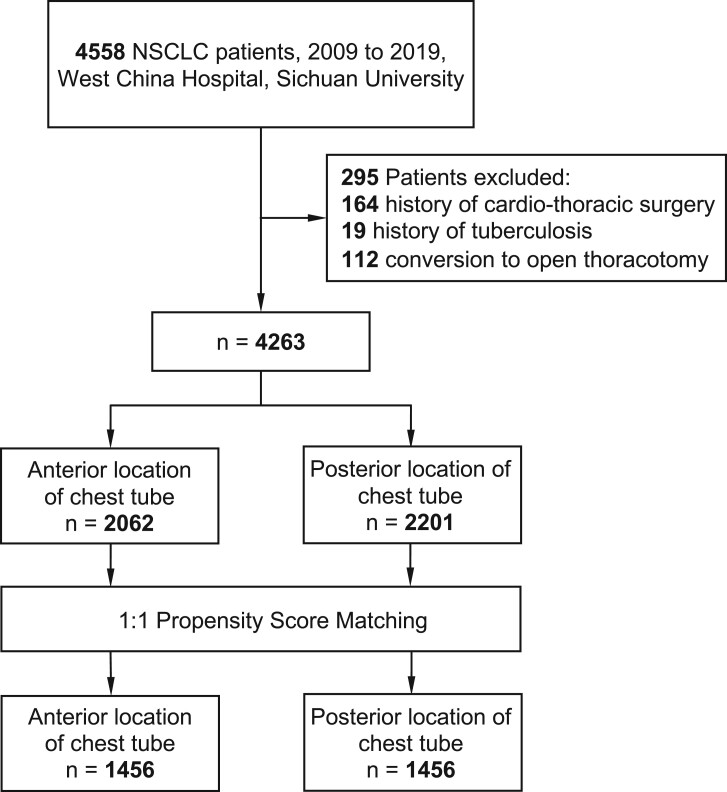
Flow chart of inclusion procedure.

**Figure 2:**
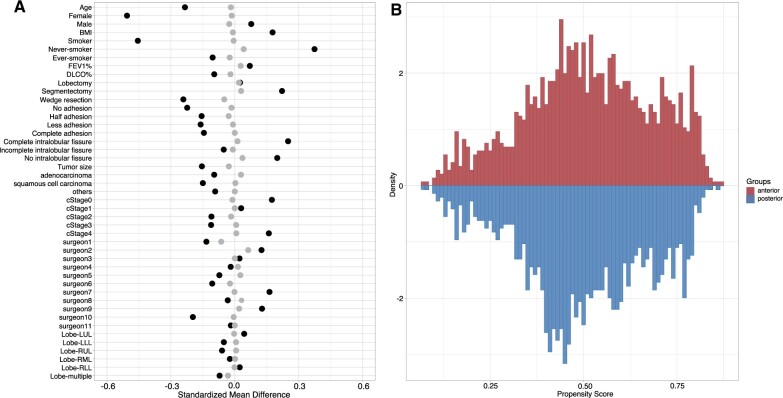
(**A**) Standardized differences of variables between anterior and posterior group. Black and grey dots represented standardized mean differences before and after matching, respectively. (**B**) Mirror histogram of propensity scores for anterior group (above the *x*-axis) and posterior group (below the *x*-axis).

During October 2009 to August 2019, 4558 patients underwent VATS and a total of 4263 patients were finally included in the cohort (Fig. 1). We divided patients into 2 groups by the position of chest tube insertion: 2062 in the anterior and 2201 in the posterior group. The 1:1 matching for 2 groups resulted in overall 2912 (1456:1456, anterior versus posterior) patients with balanced covariates (Fig. 2). Table 1 shows the comparisons of patients’ demographic characteristics and clinical features between anterior and posterior groups before and after matching.

**Table 1:** Patients’ baseline characteristics before and after matching

	Before matching				After matching			
	Anterior	Posterior	*P*-Value^a^	SMD	Anterior	Posterior	*P*-Value^b^	SMD
*N*	2062	2201			1456	1456		
Age, years	55.98 (10.75)	56.74 (10.85)	0.02	0.07	56.14 (10.91)	56.22 (10.79)	0.85	0.007
Sex				0.02				
Female	1258 (61.01)	1318 (59.88)			889 (61.06)	884 (60.71)		
Male	804 (38.99)	883 (40.12)	0.47		567 (38.94)	572 (39.29)	0.88	0.007
BMI	22.92 (2.86)	23.09 (3.05)	0.06	0.06	23.01 (2.88)	23.06 (3.01)	0.63	0.02
Smoking status			0.20	0.06			0.93	0.01
Current	117 (5.67)	151 (6.86)			91 (6.25)	86 (5.91)		
Never	1534 (74.39)	1595 (72.47)			1081 (74.24)	1084 (74.45)		
Ever	411 (19.93)	455 (20.67)			284 (19.51)	286 (19.64)		
FEV1%	105.82 (16.77)	103.68 (17.51)	<0.001	0.12	104.87 (16.55)	104.95 (16.77)	0.87	0.005
DLCO%	100.58 (16.56)	101.12 (17.43)	0.29	0.03	100.79 (16.65)	100.85 (17.14)	0.92	0.004
Resection extent			<0.001	0.21			0.86	0.02
Lobectomy	1243 (60.28)	1493 (67.83)			923 (63.39)	936 (64.29)		
Segmentectomy	614 (29.78)	455 (20.67)			370 (25.41)	358 (24.59)		
Wedge resection	205 (9.94)	253 (11.49)			163 (11.20)	162 (11.13)		
Intralobular fissure			<0.001	0.13			0.97	0.01
Complete	39 (1.89)	35 (1.59)			29 (1.99)	28 (1.92)		
Uncomplete	1315 (63.77)	1271 (57.75)			874 (60.03)	869 (59.68)		
No fissure	708 (34.34)	895 (40.66)			553 (37.98)	559 (38.39)		
Pleural adhesion			<0.001	0.17			0.94	0.02
No	826 (40.06)	706 (32.08)			525 (36.06)	520 (35.71)		
Less	884 (42.87)	1059 (48.11)			670 (46.02)	683 (46.91)		
Half	232 (11.25)	298 (13.54)			171 (11.74)	162 (11.13)		
Complete	120 (5.82)	138 (6.27)			90 (6.18)	91 (6.25)		
Tumour size	1.79 (1.10)	2.01 (1.29)	<0.001	0.19	1.86 (1.13)	1.87 (1.17)	0.69	0.02
Histology			<0.001	0.15			0.46	0.05
Adeno	1880 (91.17)	1907 (86.64)			1298 (89.15)	1296 (89.01)		
Squamous	99 (4.80)	158 (7.18)			86 (5.91)	76 (5.22)		
Others	83 (4.03)	136 (6.18)			72 (4.95)	84 (5.77)		
Pathological stage			<0.001	0.19			0.91	0.04
Stage 0	61 (2.96)	54 (2.45)			41 (2.82)	39 (2.68)		
Stage I	1714 (83.12)	1686 (76.60)			1189 (81.66)	1180 (81.04)		
Stage II	122 (5.92)	178 (8.09)			90 (6.18)	94 (6.46)		
Stage III	136 (6.60)	227 (10.31)			107 (7.35)	118 (8.10)		
Stage IV	29 (1.41)	56 (2.54)			29 (1.99)	25 (1.72)		

Data were presented as mean (SD) and incidence (proportions).

a *P*-value was calculated using Student’s *t*-test for continuous variables and Chi square test for categorical variables.

b *P*-value was calculated using paired *t*-test for continuous variables and McNemar test for categorical variables.

BMI: body mass index; DLCO%: predicted diffusion capacity of carbon oxide; FEV1%: predicted forced expiratory volume in 1 s; SD: standard deviation; CNY: Chinese Yuan; SMD: standardized mean difference.

### Analyses of postoperative outcomes after matching

#### Postoperative drainage

The outcomes of postoperative drainage variables were comparable between the 2 matched groups. and no significant difference was found in terms of the mean chest tube duration (3.39 ± 2.81 vs 3.38 ± 2.48 ml, *P* = 0.52), drainage volume during the first 3 postoperative days (526.08 ± 393.73 vs 509.60 ± 369.40 ml, *P* = 0.29) and total drainage volume (774.53 ± 918.88 vs 762.72 ± 951.69 ml, *P* = 0.66). The mean difference and its 95% CI of chest tube duration were 0.02 (−0.17, 0.20), entirely within the prespecified equivalence margin. All the compared postoperative outcomes are shown in Table 2.

**Table 2:** Outcomes between anterior and posterior group in matched cohort

	Anterior	Posterior	*P*-Value^a^
*N*	1456	1456	
Drainage volume in first 3 postoperative days	526.08 (393.73)	509.60 (369.40)	0.29
Total drainage volume	774.53 (918.88)	762.72 (951.69)	0.66
Chest tube duration	3.39 (2.81)	3.38 (2.48)	0.52
Posterior hospital stays	5.47 (3.87)	5.24 (3.03)	0.02
Hospitalization cost, CNY	51 654.57 (13 807.83)	51 297.25 (12 561.44)	0.17
Postoperative complications	87 (5.98)	106 (7.28)	0.18
Persistent drainage	173 (11.88)	207 (14.22)	0.07
PAL	66 (4.53)	75 (5.15)	0.49
Pulmonary infection	21 (1.44)	21 (1.44)	1.00
Chylothorax	14 (0.96)	15 (1.03)	1.00
Atelectasis	0 (0.00)	2 (0.14)	0.48
Emphysema	1 (0.07)	0 (0.00)	1.00
Surgical site infection	2 (0.14)	1 (0.07)	1.00

Data were presented as mean (SD) and incidence (proportions).

a *P*-value was calculated using paired *t*-test for continuous variables and McNemar test for categorical variables.

PAL: persistent air leak; SD: standard deviation.

#### Postoperative complications

The complication rate, including persistent air leak longer than 5 days (anterior = 4.53% vs posterior = 5.15%, *P* = 0.49), persistent drainage longer than 5 days (anterior = 11.88% vs posterior = 14.22%, *P* = 0.07), pulmonary infection (anterior = 1.44% vs posterior = 1.44%, *P* = 1.00) and chylothorax (anterior = 0.96% vs posterior = 1.03%, *P* = 1.00), was similar in 2 groups. Neither anterior group nor posterior group needed a secondary intervention to reinsert chest tubes.

#### Length of postoperative hospital stay and patients’ cost

The mean length of postoperative hospital stays was statistically different between the anterior group and the posterior group (5.47 ± 3.87 vs 5.24 ± 3.03 ml, *P* = 0.02). Hospitalization cost was comparable between 2 groups (51654.57 ± 13807.83 vs 51297.25 ± 12561.44 CNY, *P* = 0.17).

### Subgroup analysis and sensitivity analysis

Outcomes in subgroup analyses are shown in Supplementary Material, Tables S1–S3. In subgroups of uniportal and three-portal VATS, no significant differences were identified in chest tube duration, drainage volume, incidence of complications and postoperative hospital stays. The 95% CI of mean differences in chest tube duration was (−0.67, 0.35) and (−0.31, 0.17) in uniportal and three-portal groups, respectively. In subgroups of individual surgeons, 95% CI of mean differences in chest tube duration exceeds the equivalence limit in 6/11 surgeons, though clinical outcomes were comparable between anterior and posterior groups. In subgroups of lobectomy and sublobectomy, the 95% CI of mean of differences was within equivalence margin. IPTW analysis revealed similar results, with the 95% CI of mean of differences as (−0.11, 0.29) (Supplementary Material, Table S4).

## DISCUSSION

We aimed to compare the drainage efficiency between the anterior and posterior chest tube position in thoracic cavity. The outcomes were similar regarding chest tube duration, drainage volume during the first 3 postoperative days, the total drainage volume, the length of postoperative hospital stays, the incidence of postoperative complications and hospitalization cost. The 95% CI of mean differences of chest tube duration was within equivalence margin.

Various strategies have been applied to improve chest tube drainage to benefit rehabilitation after lung surgery, including surgery technique [13], fibrin sealants patch [14], suction or non-suction on chest tubes [9] and digital drainage system [10]. However, the optimal position inside the thoracic cavity to place chest tube has not been identified. Commonly, we inserted chest tubes in the bottom of thoracic cavity to drain excessive fluid for haemothorax and pleural effusion. And we indwelled chest tubes in the apex of thoracic cavity to drain excessive air for pneumothorax [15].

Current locations to insert chest tubes mainly included Bulau position (the 4th or 5th intercostal space in the midaxillary line) and Monaldi position (the 2nd or 3rd intercostal space in the midclavicular line) [16, 17]. However, these positions were identified based on the insertion site on the skin rather than in the thoracic cavity. The choice to place chest tubes in the anterior or posterior thoracic cavity is currently based on clinicians’ preferences and no consensus has been reached. The main difference of the position to insert chest tubes was associated with the ability to drain excessive air and fluid. We assumed that air might accumulate mostly in the anterior thoracic cavity, while effusion might be aggregated mostly in the posterior cavity due to the horizontal or semi-reclining position postoperatively. Thus, inserting a chest tube anterior to the remaining lobe might benefit air drainage, while the chest tube posterior to the remaining lobe may boost fluid drainage.

Medical literatures and textbooks recommended using 2 tubes, since they hypothesized that 2 chest tubes could deal with both the air and fluid drainage compared to a single tube [18]. However, studies showed no better clinical result using 2 chest tubes compared to 1 tube [19, 20]. Therefore, we assumed that the drainage efficiency of the anterior versus posterior insertions might be similar when using 1 tube. The results met the equivalence criteria, supporting our hypothesis. Although the postoperative hospital stays were statistically different between anterior and posterior groups, the absolute difference of 0.23 days has limited clinical significance. We concluded some explanations on our results. A single chest tube drains most of fluid and air from the apex to bottom of thoracic cavity with a number of ports on the lateral wall of the tube. With progressive lung reinflation and eliminated dead space in thoracic cavity, most air might be compressed to the posterior thoracic cavity. Assisted by postoperative coughing and changes in patients’ position, the fluid might not be aggregated in the fixed position. Thus, inserting chest tube anteriorly or posteriorly in the thoracic cavity could drain excessive air and fluid with similar efficiency. It should be free on anterior or posterior chest tube position in thoracic cavity.

A few previous studies focused on the optimal chest drain position for pneumothorax or trauma. Riber *et al.* [21] conducted a retrospective study of 134 patients with primary spontaneous pneumothorax and divided them into 3 groups according to chest tubes locations: apex, middle and basal cavity. They concluded that the location of chest tubes in the pleural cavity did not influence postoperative drainage. Benns *et al.* [22] reviewed 291 patients undergoing tube thoracotomy for trauma. They defined the chest tubes inserted above the 6th rib space as ‘high’, and the others as ‘low’. Their results concluded that chest tube position did not influence the need for secondary interventions.

On the aspect of the tube numbers, our findings supported the suggestion that it was not necessary to place 2 chest tubes, since 1 chest tube might have the ability to take care both air and fluid drainage. In 2003, Alex *et al.* [23] compared the results of single and double chest tubes; the mean total drainage was 667 ml in the double-tube group and 804 ml in the single-tube group. In 2009, Okur *et al.* [24] conducted a prospective randomized study to compare the single- and double-tube groups. The results showed that the double-tube group had the overall drainage of 896 ml, which seemed larger than that in our study. Therefore, the use of 1 tube could provide favourable clinical outcomes compared with 2 tubes in previous studies.

To our knowledge, this is the first retrospective study to identify the comparable drainage efficiency of different positions (anterior or posterior in thoracic cavity) to insert chest tube after lung cancer resection. Furthermore, we performed PSM analyses to balance baseline characteristics and followed strict inclusion and exclusion criteria to guarantee the comparative characteristics of 2 groups. We performed a number of subgroup analyses regarding uniportal versus three-portal VAT, individual surgeons and resection extent. We also performed sensitivity analysis using different matching methodology. Those additional analyses supported the robustness of our findings.

###  

We inevitably have some limitations to this study. First, the retrospective, single-institution characteristics restricted its reliability. We cannot entirely omit potential selection bias for the non-randomized study design. Second, the limited patients included in our study may reduce power to detect difference between the 2 groups. Herein, this warrants further investigations in other cohorts and prospective randomized controlled trial to further confirm the identical effect of anterior and posterior positions of chest tube after pulmonary surgery.

## CONCLUSION

For lung cancer patients undergoing VATS resection, the comparison between anterior and posterior insertion of single chest tube in the thoracic cavity met the criteria for equivalence, in terms of the outcomes in postoperative drainage and morbidities. It was free of choice on anterior or posterior single-tube insertion.

## SUPPLEMENTARY MATERIAL

Supplementary material is available at *ICVTS* online.

## Supplementary Material

ivac069_Supplementary_Data

## ABBREVIATIONS

CI: Confidence interval
IPTW: Inverse probability of treatment weight
PSM: Propensity score-matched
VATS: Video-assisted thoracoscopic surgery
