# Supplementary material for: Free of choice on anterior and posterior chest tube position after lung cancer resection
Source: Interact Cardiovasc Thorac Surg. 2022 Mar 14;35(1):ivac069. doi: 10.1093/icvts/ivac069 (PMC9387293; doi:10.1093/icvts/ivac069)
Supplement: ivac069_Supplementary_Data [file ivac069_supplementary_data.docx]

Supplementary Table 1. Outcomes in subgroup of patients undergoing uniportal and three-portal VATS.

|  | Uniportal | | | | Three-portal | | |  |  |
| --- | --- | --- | --- | --- | --- | --- | --- | --- | --- |
|  | anterior | posterior |  | anterior | | posterior |  | | |
| Variables | 130 | 154 | P^a^ | 1311 | | 1282 | ^a^ P | | |
| Chest tube duration | 3.04 (2.02) | 3.21 (2.40) | 0.526 | 3.41 (2.54) | | 3.48 (3.62) | 0.581 | | |
| 95% CI of mean of differences | (-0.67, 0.35) | |  | (-0.31, 0.17) | | |  | | |
| Drainage volume in first 3 postoperative days | 493.72 (375.12) | 475.88 (418.08) | 0.708 | 511.28 (364.11) | | 531.44 (391.54) | 0.175 | | |
| Total drainage volume | 751.43 (1062.42) | 740.81 (1375.49) | 0.943 | 753.93 (926.92) | | 771.06 (864.82) | 0.627 | | |
| Posterior hospital stays | 4.69 (2.13) | 5.08 (2.63) | 0.181 | 5.26 (3.08) | | 5.58 (4.65) | 0.039 | | |
| Hospitalization cost, CNY | 49312.67 (11598.65) | 49228.78 (10501.44) | 0.949 | 51174.78 (12444.42) | | 52099.28 (14198.32) | 0.078 | | |
| Postoperative complications | 5 (3.85) | 7 (4.55) | 1 | 89 (6.79) | | 80 (6.24) | 0.627 | | |
| Persistent drainage | 16 (12.31) | 17 (11.04) | 0.883 | 189 (14.42) | | 154 (12.01) | 0.08 | | |
| b PAL | 4 (3.08) | 7 (4.55) | 0.741 | 65 (4.96) | | 57 (4.45) | 0.601 | | |
| Pulmonary infection | 0 (0.00) | 2 (1.30) | 0.554 | 18 (1.37) | | 19 (1.48) | 0.945 | | |
| chylothorax | 2 (1.54) | 1 (0.65) | 0.883 | 10 (0.76) | | 12 (0.94) | 0.79 | | |
| atelectasis | 0 (0.00) | 0 (0.00) | ^c^ NA | 2 (0.15) | | 1 (0.08) | 1 | | |
| emphysema | 0 (0.00) | 0 (0.00) | NA | 0 (0.00) | | 2 (0.16) | 0.47 | | |
| Surgical site infection | 0 (0.00) | 0 (0.00) | NA | 1 (0.08) | | 2 (0.16) | 0.985 | | |

Data were presented as mean (SD) and incidence (proportions). ^a^ P value were calculated using Student’s t-test for continuous variables and Chi-square test for categorical variables. ^b^ Persistent air leak, PAL. ^c^ P value was unable to calculate.

Supplementary Table 2. Outcomes in subgroup of individual surgeon.

|  | Surgeon 1 | | | Surgeon 2 | | |
| --- | --- | --- | --- | --- | --- | --- |
|  | anterior | posterior |  | anterior | posterior |  |
| Variables | 130 | 154 | ^a^ P | 1311 | 1282 | ^a^ P |
| Chest tube duration | 2.19 (1.50) | 2.56 (2.22) | 0.027 | 3.68 (2.23) | 3.43 (2.66) | 0.532 |
| 95% CI of mean of differences | (-0.69, 0.04) | |  | (-0.54, 1.04) | |  |
| Drainage volume in first 3 postoperative days | 497.52 (351.09) | 515.61 (356.83) | 0.557 | 506.95 (388.98) | 507.33 (457.08) | 0.996 |
| Total drainage volume | 736.32 (877.95) | 773.43 (859.08) | 0.623 | 677.47 (726.68) | 718.67 (831.14) | 0.745 |
| Posterior hospital stays | 3.86 (2.00) | 4.43 (2.74) | 0.007 | 5.34 (1.99) | 6.52 (7.91) | 0.206 |
| Hospitalization cost, CNY | 45062.96 (9713.27) | 46437.83 (16494.84) | 0.244 | 52445.86 (10146.85) | 55397.67 (11690.02) | 0.098 |
| Postoperative complications | 4 (1.53) | 7 (2.61) | 0.568 | 7 (9.09) | 7 (9.33) | 1 |
| Persistent drainage | 12 (4.58) | 17 (6.34) | 0.483 | 16 (20.78) | 12 (16.00) | 0.582 |
| ^b^ PAL | 3 (1.15) | 4 (1.49) | 1 | 4 (5.19) | 4 (5.33) | 1 |
| Pulmonary infection | 1 (0.38) | 1 (0.37) | 1 | 0 (0.00) | 2 (2.67) | 0.465 |
| chylothorax | 0 (0.00) | 2 (0.75) | 0.489 | 1 (1.30) | 0 (0.00) | 1 |
| atelectasis | 0 (0.00) | 0 (0.00) | ^c^ NA | 0 (0.00) | 0 (0.00) | NA |
| emphysema | 0 (0.00) | 0 (0.00) | NA | 0 (0.00) | 1 (1.33) | 0.989 |
| Surgical site infection | 0 (0.00) | 0 (0.00) | NA | 0 (0.00) | 1 (1.33) | 0.989 |
|  | Surgeon 3 |  |  | Surgeon 4 |  |  |
|  | anterior | posterior |  | anterior | posterior |  |
| Variables | 19 | 22 | P | 120 | 120 | P |
| Chest tube duration | 3.11 (1.37) | 3.91 (2.60) | 0.234 | 3.24 (2.67) | 3.17 (3.36) | 0.865 |
| 95% CI of mean of differences | (-2.10, 0.49) | |  | (-0.71, 0.84) | |  |
| Drainage volume in first 3 postoperative days | 619.05 (427.00) | 519.45 (409.04) | 0.451 | 469.83 (328.21) | 521.42 (353.80) | 0.243 |
| Total drainage volume | 1415.37 (2694.59) | 659.00 (611.49) | 0.208 | 731.58 (1000.15) | 808.40 (805.74) | 0.513 |
| Posterior hospital stays | 4.84 (2.19) | 6.00 (2.81) | 0.154 | 5.11 (3.56) | 5.04 (4.20) | 0.895 |
| Hospitalization cost, CNY | 52014.20 (13048.85) | 50785.99 (10482.14) | 0.74 | 52118.60 (10882.12) | 49805.39 (10571.21) | 0.096 |
| Postoperative complications | 0 (0.00) | 4 (18.18) | 0.153 | 12 (10.00) | 6 (5.00) | 0.22 |
| Persistent drainage | 2 (10.53) | 4 (18.18) | 0.804 | 13 (10.83) | 13 (10.83) | 1 |
| ^b^ PAL | 0 (0.00) | 4 (18.18) | 0.153 | 6 (5.00) | 6 (5.00) | 1 |
| Pulmonary infection | 0 (0.00) | 0 (0.00) | NA | 3 (2.50) | 3 (2.50) | 1 |
| chylothorax | 0 (0.00) | 0 (0.00) | NA | 2 (1.67) | 1 (0.83) | 1 |
| atelectasis | 0 (0.00) | 0 (0.00) | NA | 0 (0.00) | 0 (0.00) | NA |
| emphysema | 0 (0.00) | 0 (0.00) | NA | 0 (0.00) | 0 (0.00) | NA |
| Surgical site infection | 0 (0.00) | 0 (0.00) | NA | 0 (0.00) | 0 (0.00) | NA |
|  | Surgeon 5 | | | Surgeon 6 | | |
|  | anterior | posterior |  | anterior | posterior |  |
| Variables | 66 | 79 | P | 153 | 162 | P |
| Chest tube duration | 3.09 (2.04) | 3.43 (3.14) | 0.451 | 3.09 (1.76) | 2.94 (1.87) | 0.454 |
| 95% CI of mean of differences | (-1.20, 0.52) | |  | (-0.25, 0.55) | |  |
| Drainage volume in first 3 postoperative days | 515.94 (334.61) | 476.48 (403.42) | 0.528 | 506.57 (357.19) | 523.46 (383.84) | 0.687 |
| Total drainage volume | 789.20 (929.22) | 662.56 (729.16) | 0.36 | 688.97 (644.48) | 736.71 (782.19) | 0.556 |
| Posterior hospital stays | 4.36 (1.98) | 4.76 (3.15) | 0.378 | 4.71 (2.01) | 4.95 (2.33) | 0.321 |
| Hospitalization cost, CNY | 58110.48 (11965.05) | 64445.65 (18621.50) | 0.019 | 53925.48 (11837.70) | 53228.42 (10413.25) | 0.579 |
| Postoperative complications | 0 (0.00) | 2 (2.53) | 0.557 | 9 (5.88) | 3 (1.85) | 0.116 |
| Persistent drainage | 5 (7.58) | 7 (8.86) | 1 | 15 (9.80) | 13 (8.02) | 0.721 |
| ^b^ PAL | 1 (1.52) | 1 (1.27) | 1 | 4 (2.61) | 2 (1.23) | 0.629 |
| Pulmonary infection | 0 (0.00) | 0 (0.00) | NA | 3 (1.96) | 2 (1.23) | 0.949 |
| chylothorax | 0 (0.00) | 1 (1.27) | 1 | 1 (0.65) | 0 (0.00) | 0.977 |
| atelectasis | 0 (0.00) | 0 (0.00) | NA | 0 (0.00) | 0 (0.00) | NA |
| emphysema | 0 (0.00) | 0 (0.00) | NA | 0 (0.00) | 0 (0.00) | NA |
| Surgical site infection | 0 (0.00) | 0 (0.00) | NA | 0 (0.00) | 0 (0.00) | NA |
|  | Surgeon 7 |  |  | Surgeon 8 |  |  |
|  | anterior | posterior |  | anterior | posterior |  |
| Variables | 319 | 309 | P | 185 | 171 | P |
| Chest tube duration | 4.20 (3.19) | 4.48 (4.01) | 0.341 | 3.76 (2.92) | 3.43 (6.07) | 0.509 |
| 95% CI of mean of differences | (-0.84, 0.29) | |  | (-0.68, 1.34) | |  |
| Drainage volume in first 3 postoperative days | 508.29 (384.52) | 550.09 (423.35) | 0.195 | 471.57 (325.32) | 484.49 (343.15) | 0.716 |
| Total drainage volume | 718.54 (864.26) | 838.85 (1206.87) | 0.15 | 668.90 (826.57) | 640.81 (619.57) | 0.719 |
| Posterior hospital stays | 6.27 (3.52) | 6.92 (5.16) | 0.064 | 5.39 (3.20) | 5.22 (6.36) | 0.744 |
| Hospitalization cost, CNY | 52959.33 (15119.65) | 53218.68 (14392.11) | 0.826 | 49283.42 (12712.46) | 48826.42 (10048.77) | 0.708 |
| Postoperative complications | 35 (10.97) | 40 (12.94) | 0.523 | 14 (7.57) | 8 (4.68) | 0.362 |
| Persistent drainage | 75 (23.51) | 66 (21.36) | 0.582 | 34 (18.38) | 13 (7.60) | 0.004 |
| ^b^ PAL | 34 (10.66) | 36 (11.65) | 0.789 | 11 (5.95) | 5 (2.92) | 0.263 |
| Pulmonary infection | 6 (1.88) | 7 (2.27) | 0.954 | 1 (0.54) | 1 (0.58) | 1 |
| chylothorax | 3 (0.94) | 4 (1.29) | 0.966 | 3 (1.62) | 2 (1.17) | 1 |
| atelectasis | 0 (0.00) | 0 (0.00) | NA | 2 (1.08) | 0 (0.00) | 0.513 |
| emphysema | 0 (0.00) | 0 (0.00) | NA | 0 (0.00) | 1 (0.58) | 0.969 |
| Surgical site infection | 1 (0.31) | 0 (0.00) | 1 | 0 (0.00) | 0 (0.00) | NA |
|  | Surgeon 9 | | | Surgeon 10 | | |
|  | anterior | posterior |  | anterior | posterior |  |
| Variables | 150 | 141 | P | 42 | 44 | P |
| Chest tube duration | 3.55 (2.13) | 3.55 (2.44) | 0.981 | 4.02 (2.53) | 3.43 (1.26) | 0.171 |
| 95% CI of mean of differences | (-0.54, 0.52) | |  | (-0.28, 1.46) | |  |
| Drainage volume in first 3 postoperative days | 593.07 (410.85) | 562.65 (439.15) | 0.542 | 639.88 (403.13) | 602.84 (485.26) | 0.702 |
| Total drainage volume | 984.29 (1217.26) | 836.26 (987.68) | 0.257 | 1026.79 (984.59) | 980.23 (1508.75) | 0.867 |
| Posterior hospital stays | 5.67 (3.33) | 5.62 (2.78) | 0.891 | 7.24 (3.69) | 6.25 (2.39) | 0.143 |
| Hospitalization cost, CNY | 50773.49 (10803.06) | 52586.82 (11997.67) | 0.176 | 54913.52 (10523.14) | 52361.85 (8643.25) | 0.222 |
| Postoperative complications | 11 (7.33) | 7 (4.96) | 0.552 | 3 (7.14) | 0 (0.00) | 0.224 |
| Persistent drainage | 24 (16.00) | 16 (11.35) | 0.326 | 9 (21.43) | 2 (4.55) | 0.043 |
| ^b^ PAL | 5 (3.33) | 3 (2.13) | 0.787 | 1 (2.38) | 0 (0.00) | 0.981 |
| Pulmonary infection | 3 (2.00) | 3 (2.13) | 1 | 1 (2.38) | 0 (0.00) | 0.981 |
| chylothorax | 1 (0.67) | 1 (0.71) | 1 | 2 (4.76) | 0 (0.00) | 0.454 |
| atelectasis | 0 (0.00) | 1 (0.71) | 0.975 | 0 (0.00) | 0 (0.00) | NA |
| emphysema | 0 (0.00) | 0 (0.00) | NA | 0 (0.00) | 0 (0.00) | NA |
| Surgical site infection | 0 (0.00) | 0 (0.00) | NA | 0 (0.00) | 1 (2.27) | 1 |
|  | Surgeon 11 | | |  |  |  |
|  | anterior | posterior |  |  |  |  |
| Variables | 63 | 65 | P |  |  |  |
| Chest tube duration | 3.21 (1.49) | 3.72 (2.47) | 0.156 |  |  |  |
| 95% CI of mean of differences | (-1.23, 0.20) | |  |  |  |  |
| Drainage volume in first 3 postoperative days | 464.92 (304.04) | 529.12 (368.84) | 0.285 |  |  |  |
| Total drainage volume | 575.56 (505.21) | 695.60 (650.79) | 0.247 |  |  |  |
| Posterior hospital stays | 4.84 (1.77) | 5.45 (3.49) | 0.22 |  |  |  |
| Hospitalization cost, CNY | 53623.61 (12154.29) | 53772.17 (10983.62) | 0.942 |  |  |  |
| Postoperative complications | 1 (1.59) | 5 (7.69) | 0.224 |  |  |  |
| Persistent drainage | 5 (7.94) | 11 (16.92) | 0.204 |  |  |  |
| ^b^ PAL | 1 (1.59) | 1 (1.54) | 1 |  |  |  |
| Pulmonary infection | 0 (0.00) | 2 (3.08) | 0.49 |  |  |  |
| chylothorax | 0 (0.00) | 2 (3.08) | 0.49 |  |  |  |
| atelectasis | 0 (0.00) | 0 (0.00) | NA |  |  |  |
| emphysema | 0 (0.00) | 0 (0.00) | NA |  |  |  |
| Surgical site infection | 0 (0.00) | 0 (0.00) | NA |  |  |  |

Data were presented as mean (SD) and incidence (proportions). ^a^ P value were calculated using Student’s t-test for continuous variables and Chi-square test for categorical variables. ^b^ Persistent air leak, PAL. ^c^ NA, P value was unable to calculate.

Supplementary Table 3. Outcomes in subgroup of patients undergoing lobectomy and sublobectomy (segmentectomy and wedge resection).

|  | Lobectomy | | | | Sublobectomy | | | | | |
| --- | --- | --- | --- | --- | --- | --- | --- | --- | --- | --- |
|  | anterior | posterior | ^a^ P | | anterior | | posterior | | P | |
| Variables | 923 | 936 |  | | 533 | | 520 | |  | |
| Chest tube duration | 3.69 (2.60) | 3.83 (3.30) | 0.289 | | 2.85 (2.23) | | 2.75 (3.72) | | 0.618 | |
| 95% CI of mean of differences | (-0.42, 0.12) | | |  | | (-0.28, 0.47) | |  | |  |
| Drainage volume in first 3 postoperative days | 528.57 (374.92) | 526.34 (380.61) | 0.899 | | 479.77 (343.27) | | 525.19 (418.87) | | 0.054 | |
| Total drainage volume | 799.04 (979.31) | 771.38 (860.72) | 0.518 | | 674.93 (850.59) | | 764.24 (1043.34) | | 0.128 | |
| Posterior hospital stays | 5.64 (3.19) | 6.01 (4.61) | 0.046 | | 4.47 (2.50) | | 4.65 (4.02) | | 0.395 | |
| Hospitalization cost, CNY | 53277.68 (12682.35) | 54297.32 (14660.32) | 0.109 | | 47486.03 (11563.20) | | 47295.71 (10947.72) | | 0.784 | |
| Postoperative complications | 77 (8.34) | 74 (7.91) | 0.795 | | 19 (3.56) | | 15 (2.88) | | 0.653 | |
| Persistent drainage | 165 (17.88) | 148 (15.81) | 0.26 | | 45 (8.44) | | 26 (5.00) | | 0.035 | |
| ^b^ PAL | 55 (5.96) | 57 (6.09) | 0.983 | | 15 (2.81) | | 9 (1.73) | | 0.331 | |
| Pulmonary infection | 16 (1.73) | 19 (2.03) | 0.765 | | 2 (0.38) | | 2 (0.38) | | 1 | |
| chylothorax | 10 (1.08) | 10 (1.07) | 1 | | 3 (0.56) | | 3 (0.58) | | 1 | |
| atelectasis | 2 (0.22) | 1 (0.11) | 0.99 | | 0 (0.00) | | 0 (0.00) | | NA | |
| emphysema | 0 (0.00) | 1 (0.11) | 1 | | 0 (0.00) | | 1 (0.19) | | 0.99 | |
| Surgical site infection | 1 (0.11) | 1 (0.11) | 1 | | 0 (0.00) | | 1 (0.19) | | 0.99 | |

Data were presented as mean (SD) and incidence (proportions). ^a^ P value were calculated using Student’s t-test for continuous variables and Chi-square test for categorical variables. ^b^ Persistent air leak, PAL. ^c^ P value was unable to calculate.

Supplementary Table 4. Outcomes between anterior and posterior group after IPTW matching.

|  | anterior | posterior |  |
| --- | --- | --- | --- |
| Variables | 4278.5 | 4222.2 | ^a^ P |
| Chest tube duration | 3.49(2.61) | 3.40(2.91) | 0.388 |
| 95% CI of mean of differences | (-0.11, 0.29) | |  |
| Drainage volume in first 3 postoperative days | 511.25(371.84) | 525.41(393.72) | 0.327 |
| Total drainage volume | 766.57(1027.99) | 762.54(929.99) | 0.914 |
| Posterior hospital stays | 5.33(3.19) | 5.47(4.00) | 0.326 |
| Hospitalization cost, CNY | 51698.67(11842.88) | 51079.91(13930.42) | 0.212 |
| Postoperative complications | 257.4(6.0) | 266.7(6.3) | 0.702 |
| Persistent drainage | 522.8(12.2) | 572.1(13.6) | 0.234 |
| ^b^ PAL | 199.4(4.7) | 184.4(4.4) | 0.663 |
| Pulmonary infection | 56.7(1.3) | 53.8(1.3) | 0.893 |
| chylothorax | 37.7(0.9) | 32.5(0.8) | 0.694 |
| atelectasis | 2.5(0.1) | 4.8(0.1) | 0.464 |
| emphysema | 3.4(0.1) | 0.0(0.0) | 0.166 |
| Surgical site infection | 8.6(0.2) | 1.3(0.0) | 0.078 |

Data were presented as mean (SD) and incidence (proportions). ^a^ P value were calculated using Student’s t-test for continuous variables and Chi-square test for categorical variables. ^b^ Persistent air leak, PAL. ^c^ P value was unable to calculate.
